# Is Oocyte Quality Impaired in Cases With Ovarian Endometriosis? A Second Look Into the Clinical Setting

**DOI:** 10.3389/fendo.2022.921032

**Published:** 2022-06-30

**Authors:** Johnny S. Younis

**Affiliations:** ^1^ Reproductive Medicine, Department of Obstetrics and Gynecology, Baruch-Padeh Medical Center, Poriya, Israel; ^2^ Azrieili Faculty of Medicine in Galilee, Safed, Bar-Ilan University, Safed, Israel

**Keywords:** endometriosis, endometrioma, oocyte quality, oocyte competence, morphokinetics, vitrification

## Introduction

Endometriosis is a worldwide, widespread, chronic, and inflammatory gynecological disease that affects 1 in 10 women during their reproductive years. Up to 50% of women with endometriosis have infertility. However, the possible pathophysiologic mechanisms leading to endometriosis-associated infertility remain poorly comprehended, explicitly in cases where no apparent mechanical factor is involved. Reduced fecundity in patients with endometriosis has been related to a wide variety of reasons acting at the level of the pelvic cavity, ovaries, and the uterus (1, 2). In addition to pelvic adhesions, other mechanisms were suggested, including pelvic inflammation, increased oxidative stress, dysregulation of the immune system, disturbed folliculogenesis, ovulatory dysfunction, and defective implantation.

Ovarian endometriosis, specifically endometriotic cyst or endometrioma, is a distinctive variation of the disease identified in up to 44% of affected women (3). It represents the most pathognomonic and diagnosed form due to ultrasound technology advancement (4). Due to its anatomical locus, ovarian endometriosis may negatively impact folliculogenesis and oocyte competence, possibly more than the disease’s superficial or deep infiltrating forms. Therefore, endometrioma per se may impair oocyte quality and lead to deficient fertilization, sub-optimal embryo development, and flawed implantation. Furthermore, studying separately the impact of different clinical forms of endometriosis may reveal new insights and pave the way for understanding the composite mechanisms leading to low fecundity and endometriosis-associated infertility.

Although *in-vitro* and basic science studies have suggested mechanisms that may explain endometriosis-associated oocyte quality and embryo development impairment, the evidence is inconclusive (5, 6). In the clinical setting, comparable rates of blastocyst aneuploidy were reported in a large series of women with endometriosis equivalent to their age-matched peers of IVF controls, implying that spindle apparatus alterations and oocyte meiotic errors are not implicated in endometriosis-associated infertility (7). Therefore, other oocyte-related pathophysiological mechanisms should be explored.

Assisted reproductive technologies (ART) is an impeccable and convenient clinical setting where inquiries into oocyte quality and development in endometriosis cases and its association to pregnancy achievement could be investigated in well-planned and targeted studies. The following perspective examines contemporary clinical evidence available to evaluate oocyte quality in patients with endometriosis undergoing ART treatment. Studies that assess ovarian endometriosis on oocyte competence will be emphasized. Numerous aspects of oocyte quality or impacted by oocyte competence will be explored, including morphology, embryology, morphokinetics, survival following vitrification, clinical pregnancy, and live birth rates.

To properly achieve this objective, a search of the published English literature was done on Pubmed.com from January 2011 to December 2021, with the keywords ‘endometriosis’, ‘endometrioma’, ‘ovarian endometriosis’, ‘IVF’, ‘ICSI’, ‘oocyte morphology’, ‘oocyte maturation’, ‘fertilization’, ‘top quality embryo’, blastulation’, ‘time-lapse’, ‘morphokinetics’, ‘oocyte vitrification’, ‘clinical pregnancy, ‘live birth’ and meta-analysis. The relevance of attained publications was evaluated following reading the abstract. A manual search of review articles and cross-references completed the search. Articles with an unsuitable design were excluded.

## Oocyte Morphology

Few studies in the ART setting recently targeted oocyte morphology in women with endometriosis with contrasting results (8–10). All three published studies had a retrospective and controlled design (**Table 1**). None targeted women exclusively with ovarian endometriosis, although some cases had a verified endometrioma (9, 10). Of interest and most comprehensive is the study of Robin et al, published recently, that targeted oocyte morphology as the primary outcome measure on large oocyte cohorts. Furthermore, it comprised a thorough oocyte morphology evaluation to detect a wide range of abnormalities relying on validated scores. The researchers investigated 175 women with endometriosis and 401 controls and evaluated 2016 and 4073 oocytes, respectively (10). Among women with endometriosis, 48% had an endometrioma. In this study, the average oocyte quality index and metaphase II oocyte morphological scoring system was not different, refuting the negative impact of endometriosis on oocyte morphology in the ART setting.

**Table 1:** Oocyte quality and embryo development in infertile women with endometriosis as compared to controls.

Study	Study design	No of women	Age* (years)	No of cycles	No of oocytes	Ovarian endometriosis	Oocyte morphology	Oocyte maturation rate	Fertilization rate	Top-quality embryos	Blastulation rate
		Study control		Study Control	Study Control						
**Coccia et al., 2011** (11)	Retrospective matched controlled	148 72	<35	167 80		ND 64% stage 3-4			No change		
**Benaglia et al., 2013** (12)	Retrospective multicenter cohort	39 78	36.4±3.2	39 78		All bilateral endometrioma 23±10 mm			No change	No change	
**Filippe et al., 2014** (13)	Prospective cohort	29 Sibling oocytes	35.9±4.0	29	97 103	All unilateral endometrioma 25±9 mm		No change	No change	No change	
**Shebl et al., 2017** (8)	Retrospective matched case-control	114 119	33.2±4.4	129 129	1143 1200	ND 41% stage 3-4	lower	lower	No change	No change	No change
**Kasapoglu et al., 2018** (9)	Retrospective matched control	72 60	30.9±3.9	72 60	775 793	Part with endometrioma	lower	No change	higher		
**Muteshi et al., 2018** (14)	Retrospective cohort controlled	531 737	Median 35	531 737		ND 52% stage 3-4		lower	lower		lower
**Boucret et al., 2020** (15)	Retrospective cohort controlled	84 590	32.1±3.5	155 969		ND 82% stage 3-4		No change		No change	
**Li et al., 2020** (16)	Retrospective Cohort controlled	459 360	31.5±3.5	459 360		79% endometrioma 21% superficial		No change	No change	No change	
**Pacchiarotti et al., 2020** (17)	Retrospective cohort	50 84	35.0±2.0	48 74		ND All stage 4		lower		No change	
**Sanchez et al., 2020** (18)	Retrospective matched control	309 766	36.9±3.6	429 851	2531 4936	ND All stages 3-4		No change	No change	No change	No change
**Robin et al., 2021** (10)	Retrospective cohort	175 401	31.4±3.6	348 576	2016 4073	48% endometrioma	No change	No change	No change	lower	
**Wu et al., 2021** (19)	Retrospective matched control	862 862	32.7±4.5	862 862		All endometrioma 21.6±9.7 mm		lower	No change	No change	lower

*mean age of women with endometriosis.

ND, not disclosed.

References 8, 19, and 10 include data relevant to oocyte morphology and embryo development.

## Embryo Development

Searching the literature for contemporary studies that targeted embryo development in women with endometriosis undergoing IVF treatment revealed 12 such studies published in the last decade (8–19). All but one study (13) were retrospective in design and were controlled. However, their results concerning oocyte maturation, fertilization, top-embryo quality, and blastulation were contrasted (**Table 1**). This inconsistency may highlight the controversy in the clinical setting regarding the contribution of oocyte quality and competence to the pathophysiological mechanisms of endometriosis-associated infertility.

Among the 12 studies, three targeted only women with ovarian endometrioma (12, 13, 19). Filippe et al. in a prospective study including 29 women with an intact endometrioma of 25 ± 9 mm, studying sibling oocytes, did not find an adverse impact on oocyte maturation, fertilization, and top embryo quality rates (13). Similarly, Benaglia et al, in a retrospective study of 39 women with intact bilateral endometrioma of a mean diameter of 23 ± 10 mm, did not find an adverse impact on oocyte quality, fertilization rate, or top embryo rate compared to controls (12).

Conversely, a recent retrospective study that evaluated infertile women with an endometrioma of 22 ± 10 mm matched to controls, each comprising 826 cases, showed lower maturation and blastulation rates, though similar fertilization and top embryo quality rates in the study group as compared to controls (19). Of notice, women with an endometrioma included were before (n=293) or after (n=569) endometriotic cystectomy. Women following ovarian surgery showed better oocyte maturation, fertilization, and top-quality embryo rates. Nevertheless, multivariate logistic regression analysis showed no correlation between endometrioma per se and live birth after adjusting for the number of top-quality embryos transferred and embryo transfer stage.

Another study by Li et al. retrospectively designed deserves special attention since a unique comparison was made between ovarian (n=363) and superficial (n=96) endometriosis on embryo development (16). Although comparable maturation, fertilization, cleavage, and high-quality embryo rates were found between endometriosis patients (n=459) and controls (n=360), these same parameters were superior in the ovarian compared to the superficial endometriosis cases, suggesting that the peritoneal form of the disease is responsible for the oocyte quality impairment (16).

Collectively, most data on oocyte fertilization and development in cases with ovarian endometriosis come from retrospective controlled studies that may contain several confounders, such as the size and laterality of endometrioma and ovarian surgery, which preclude definite conclusions. Available evidence from one prospective and a few retrospective studies suggest that oocyte quality is not impaired in cases with ovarian endometriosis. Furthermore, low-level evidence suggests that oocyte quality impairment may be attributed to superficial endometriosis. Prospective targeted studies are to be performed to corroborate these findings.

## Time-Lapse Morphokinetics

Time-lapse morphokinetic analysis may predict embryo development, implantation, and live birth in the ART setting, minimizing environmental influences (20–22), rendering this technology potentially a surrogate measure of oocyte quality. However, to the best of our knowledge, only five low-scale reports (23–27), four retrospective, and one prospective (n=20-126) have been published so far, examining morphokinetics in women with endometriosis compared to controls and reaching contrasting results (**Table 2**). While some found morphokinetic delay in embryos developing from endometriosis women than controls (24, 25, 27), suggesting a poorer oocyte quality, others did not (23, 26). Of interest, one prospective observational study that targeted ovarian endometriosis examined sibling oocytes in twenty women with an endometrioma (23). Sixty-nine retrieved from an ovary with an intact endometrioma compared to 59 embryos from the contralateral normal ovary showed no significant change in morphokinetic parameters.

**Table 2:** Summary of clinical studies evaluating embryos by time-lapse morphokinetics in endometriosis patients as compared to controls.

Study	Design	Number of women	Age (years)*	Number of embryos	Ovarian endometriosis	Morphokinetics
		Study Control		Study Control		
**Demirel et al., 2016** (23)	Prospective observational	20 Sibling oocytes	32.0±3.3	69 59 Sibling oocytes	No change in kinetics
**Freis et al., 2018** (24)	Retrospective cohort	72 96	34.2±ND	213 264	ND 33% stages 3-4	Altered kinetics
**Boynukalin et al., 2019** (25)	Retrospective case-control	53 29	31.9±2.7	264 175	Part with endometrioma All stages 3-4	Early kinetics affected
**Schenk et al., 2019** (26)	Retrospective cohort	86 77	32.1±ND	552 596	ND 62% stages 3-4	No change in kinetics; accelerated early cell division synchronization
**Llarena et al., 2021** (27)	Retrospective controlled	126 233	33.7±3.7	1078 2393	Part with endometrioma 56% stages 3-4	Early and late kinetics impaired

*mean age of women with endometriosis.

ND, not disclosed.

Altogether, few studies in the ART setting were published examining the impact of endometriosis on embryo development morphokinetics, including a few cases and reaching contrasting results. In addition, only one small-scale prospective study examined exclusively ovarian endometriosis with no impact on embryo kinetics and presumably oocyte quality. Therefore, further targeted studies should be conducted to examine oocyte quality in this model.

## Oocyte Survival Following Vitrification

Few cohort studies on oocyte vitrification in women with endometriosis have been published (**Table 3**), all retrospectively conducted (28–34). It seems that the most comprehensive comes from two centers of the IVI group in Spain (30, 32), especially due to the relatively high number of cases (n=1044) and high rate of women coming back (43%) to thaw their gametes. All women in this study had established ovarian endometriosis with an endometrioma > 1 cm in diameter. Other inclusion criteria were age < 42 years, AMH > 0.5 ng/mL, and AFC > 3.

**Table 3:** Oocyte vitrification and warming in cases with endometriosis.

Study	Design	Number of women	Age^&^ (years)	Ovarian endometriosis	Women returned
**Garcia-Velasco et al, 2013** (28)	Retrospective multicenter observational	38	ND	ND	5
**Raad et al, 2018** (29)	Retrospective observational cohort	49 (70 cycles)	33.9± 4.5	71% with endometrioma	ND
**Cobo et al, 2020#** (30)	Retrospective observational cohort	1044	35.7± 3.7	all women with endometrioma > 1cm	485
**Kim et al., 2020** (31)	Retrospective cohort	34	30.7±5.9	all women with endometrioma 6.0±2.5 cm	ND
**Cobo et al, 2021#** (32)	Retrospective observational cohort	1044	35.7± 3.7	all women with endometrioma > 1cm	485
Hong et al., 2021* (33)	Retrospective cohort	62 (95 cycles)	median 32.5 [27.3 - 37.8]	all women with endometrioma median 5.2 [3.3 - 6.6] cm	ND
Santulli et al., 2021* (34)	Retrospective observational cohort	146 (258 cycles)	31.5±4.4	73% with endometrioma	ND

& mean age at vitrification.

*including embryo freezing.

^#^same cohort.

ND, not disclosed.

Oocyte survival following vitrification and worming could be considered a surrogate measure of oocyte quality. Elective oocyte preservation for age-related fertility decline (social freezing) supports this notion. The survival rate of vitrified oocytes is significantly higher in women ≤35 (n=123) as compared to women >35 years of age (n=518), corresponding to 91.4% and 82.1%, respectively (35). However, in the same ART setting employing historical controls, women ≤35 years of age with endometriosis (n=260) had a significantly lower oocyte survival rate as compared to the same age groups of oocyte donors (n=15,899) or social freezing (n=123), corresponding to 85.1%, 92.3%, and 91.4%, respectively (30, 35).

Collectively, available evidence comes mainly from one complex of ART centers summarizing their clinical practice retrospectively. Within the vitrification model, oocyte quality in patients with endometriosis, all with ovarian endometriosis, seems slightly impaired, especially in women below 35 years. Further data from additional centers are required to substantiate these findings.

## Clinical Pregnancy and Live Birth Rates

Clinical pregnancy and live birth rates are complex and multifactorial events that are not considered direct oocyte quality and competence markers. Nevertheless, they are impacted by and interrelated to oocyte quality, fertilization, and development. Upraised clinical pregnancy and live births may be regarded as the eventual goals of high-quality oocytes in the ART setting.

Several systematic reviews and meta-analyses were published in the last decade examining the impact of ovarian endometriosis, specifically endometriotic cyst, on clinical pregnancy and live-birth rates (36–42). Generally, two methodologies were implemented in these studies (**Table 4**). The first compares women with an intact endometrioma to women with a normal ovary or no endometrioma (36–38). The second compares women with an intact endometrioma to women following endometriotic cystectomy (36, 39–41). Both methodologies in the ART setting showed comparable clinical pregnancy and live birth rates in all published systematic reviews and meta-analyses. These results imply that oocyte quality from an ovary with an intact endometrioma is similar to that retrieved from a normal ovary (or an ovary with no endometrioma). Furthermore, endometriotic cystectomy does not seem to improve oocyte quality compared to oocytes retrieved from an ovary with an intact endometrioma. Nonetheless, it is essential to note that most eligible reports pooled into these published systematic reviews and meta-analyses originated from retrospectively designed studies dipping their level of evidence.

**Table 4:** Summary of meta-analyses examining the impact of an intact endometrioma and endometriotic cystectomy on clinical pregnancy and live birth rates.

	Methodological design	Eligible studies’ design	Clinical pregnancy rate	Live birth rate
**Hamdan et al., 2015** (36)	Intact endometrioma versus normal ovary or no endometrioma	Prospective and retrospective	no change	no change
**Yang et al.,** **2015** (37)	Intact endometrioma versus normal ovary or no endometrioma	Prospective and retrospective	no change	no change
**Alshehre et al., 2021** (38)	Intact endometrioma versus normal ovary or no endometrioma	Prospective and retrospective	no change	no change
**Tsoumpo et al., 2009** (39)	Intact endometrioma versus cystectomy	Prospective and retrospective	no change	no change
**Hamdan et al., 2015** (36)	Intact endometrioma versus cystectomy	Prospective and retrospective	no change	no change
**Laursen et al., 2017** (40)	Intact endometrioma versus cystectomy	Prospective and retrospective	no change	no change
**Nickkho−Amiry et al., 2018** (41)	Intact endometrioma versus cystectomy	Prospective and retrospective	no change	no change
**Tao et al.,** **2017** (42)	Cystectomy versus intact endometrioma or normal ovary (combined)	Prospective and retrospective	no change	no change

Taken together, clinical pregnancy and live birth rates do not seem to be compromised in cases with an intact endometrioma nor improved following endometriotic cystectomy, suggesting that oocyte quality in ovarian endometriosis cases is not impaired within the ART setting. However, since most evidence originates from retrospective studies, further prospective targeted studies need to increase the level of evidence.

## Discussion

Most available studies on oocyte quality target endometriosis as a single distinct disorder with no differentiation between ovarian endometriosis and other forms of the disease. While it is challenging to target exclusively ovarian endometriosis, there is no conclusive evidence within the clinical setting to show that endometrioma per se impairs oocyte quality. Available current literature in cases with an intact endometrioma does not establish an adverse impact on oocyte morphology, embryo development, and morphokinetics. Although there is some data for slight oocyte quality impairment in the vitrification worming model, available evidence relies on retrospective historical controls. Furthermore, the latest systematic reviews and meta-analyses do not provide evidence to support the notion that endometrioma may harm oocyte quality since neither an intact endometrioma (36–38) nor endometriotic cystectomy (36, 39–41) impact clinical pregnancy rate and live birth rates in the ART setting.

Although *in vivo* studies and basic research has suggested mechanisms for oocyte quality impairment in women with ovarian endometriosis, most studies in the clinical ART setting, reviewed in this second look appraisal, do not substantiate these findings. One possible explanation might be that the *in-vivo* reduced fecundity in patients with endometriosis results from the toxic pelvic cavity encountered in these cases. The ART setting may neutralize this adverse influence (43, 44). It is also possible that other forms of the disease, probably superficial endometriosis, could contribute more to the toxic pelvic environment leading to natural fertility dysfunction and infertility.

Since most outcomes encountered and presented in this opinion paper rely on retrospective studies, including available systematic reviews, prospective, targeted, and well-controlled studies are essential to substantiate these findings and increase the level of evidence. Furthermore, while some studies included women following ovarian surgery, others excluded them, which should also be accounted for. Future studies, preclinical and clinical, should target different forms of the disease, especially cases with ovarian and superficial endometriosis and their direct impact on oocyte quality and competence. This strategy may allow a more comprehensiveness of the disease’s pathophysiology, which may have broader implications for targeted treatment.

## Author Contributions

The author contributed to the conception and design of the manuscript, acquisition of data, analyses, and interpretation, drafted the article, and approved it.

## Conflict of Interest

The authors declares that the research was conducted in the absence of any commercial or financial relationships that could be construed as a potential conflict of interest.

## Publisher’s Note

All claims expressed in this article are solely those of the authors and do not necessarily represent those of their affiliated organizations, or those of the publisher, the editors and the reviewers. Any product that may be evaluated in this article, or claim that may be made by its manufacturer, is not guaranteed or endorsed by the publisher.
